# Early Transcutaneous Tibial Nerve Stimulation Acutely Improves Lower Urinary Tract Function in Spinal Cord Injured Rats

**DOI:** 10.1089/neur.2021.0058

**Published:** 2022-02-14

**Authors:** Andrea M. Sartori, Souzan Salemi, Anna-Sophie Hofer, Valentin Baumgartner, Daniel Eberli, Martina D. Liechti, Martin E. Schwab, Thomas M. Kessler

**Affiliations:** ^1^ Institute for Regenerative Medicine, University of Zürich, and Department of Health Sciences and Technology, ETH Zürich, Zürich, Switzerland.; ^2^ Department of Neuro-Urology, Balgrist University Hospital, University of Zürich, Zürich, Switzerland.; ^3^ Laboratory for Tissue Engineering and Stem Cell Therapy, Department of Urology, University Hospital Zürich, Zürich, Switzerland.

**Keywords:** C-fiber bladder afferents, neurogenic lower urinary tract dysfunction (NLUTD), spinal cord injury, tibial nerve stimulation

## Abstract

Despite the fact that a majority of patients with an injury to the spinal cord develop lower urinary tract dysfunction, only few treatment options are available currently once the dysfunction arises. Tibial nerve stimulation has been used in pilot clinical trials, with some promising results. Hence, we investigated whether the early application of transcutaneous tibial nerve stimulation in the animal model of spinal cord injured rats can prevent the development of detrusor overactivity and/or detrusor-sphincter-dyssynergia. Rats were implanted with a bladder catheter and external urethral sphincter electromyography electrodes. A dorsal over-hemisection, resulting in an incomplete spinal cord injury at the T8/9 spinal level, induced immediate bladder paralysis. One week later, the animals received daily tibial nerve or sham stimulation for 15 days. Effects of stimulation on the lower urinary tract function were assessed by urodynamic investigation. Measurements showed improvements of several key parameters of lower urinary tract function—in particular, non-voiding bladder contractions and intravesical pressure—immediately after the completion of the stimulation period in the stimulated animals. These differences extinguished one week later, however. In the dorsal horn of the lumbosacral spinal cord, a small significant increase of the density of C-fiber afferents layers I-II was found in the stimulated animals at four weeks after spinal cord injury. Tibial nerve stimulation applied acutely after spinal cord injury in rats had an immediate beneficial effect on lower urinary tract dysfunction; however, the effect was transitory and did not last over time. To achieve more sustainable, longer lasting effects, further studies are needed looking into different stimulation protocols using optimized stimulation parameters, timing, and treatment schedules.

## Introduction

In the first weeks and months after spinal cord injury (SCI), progressively developing neurogenic lower urinary tract dysfunction is one of the most frequent consequences.^1,2^ Once the very acute phase after injury is over, in which the detrusor is acontractile, the bladder reflex starts to reappear but often assumes a pathological pattern: while the progressive filling activates detrusor contraction, the external urethral sphincter (EUS), instead of relaxing, also contracts, thus causing detrusor-sphincter-dyssynergia (DSD), which can be life-threatening because of urine reflux and kidney damage.^1^ In addition to DSD, neurogenic detrusor overactivity—i.e. frequent and strong involuntary detrusor contractions—often leads to urinary incontinence episodes.^3^

Standard therapy for detrusor overactivity is use of antimuscarinics, which unfortunately can also cause a considerable amount of adverse events. Therefore, alternatives and preferably even preventive treatment options are needed urgently.

Neuromodulatory therapies are emerging alternatives that involve electrical stimulation of either peripheral nerves or the central nervous system. Tibial nerve stimulation, a minimally invasive therapy, is one of them and was reported to be effective to treat idiopathic overactive bladder.^4,5^ The tibial nerve is part of the sciatic nerve, which originates in the lower lumbar levels of the spinal cord. Similarly, bladder afferent nerve fibers innervate the L4-S3 levels of the spinal cord.

To date, the mechanisms driving the positive effects of tibial nerve stimulation are still poorly understood. It has been postulated that the post-stimulation inhibitory effects are driven at the central as well as peripheral level. The inhibitory effects might derive from an activation of B3-adrenoceptors in the detrusor,^6,7^ similarly to what is observed after pudendal nerve stimulation,^8,9^ as well as central changes concerning desensitization of bladder afferent fibers of type C, neurotransmitter release,^10^ or somatosensory cortical structures.^11^ In addition to idiopathic overactive bladder, tibial nerve stimulation appears to be promising in mitigating urinary dysfunction in a variety of neurological disorders as well.^12,13^

Thus, in a reverse translation approach mirroring the situation in humans, we investigated whether the early application of transcutaneous tibial nerve stimulation suppresses the development of detrusor overactivity in a well characterized animal model of rats with large, but incomplete, spinal cord transections in the lower thoracic region. The lesion mimics severe human spinal cord injuries (American Spinal Injury Association Impairment Scale grade A to severe C) and produces locomotor and autonomic paralysis in the first week after injury, followed by minimal functional recoveries.

## Methods

### Animals

A total of 20 adult female Lewis rats were investigated in this study (LEW/OrlRj [Lewis]; 200-230 g; Janvier, France). Food (rat chow) and water were provided *ad libitum*. Rats were maintained on a 12:12 h light:dark cycle. All animals were tamed by daily handling and accustomed to the urodynamic measurement setup. All experimental procedures were conducted in accordance with ethical guidelines and were approved by the Veterinary Office of the Canton of Zurich, Switzerland.

### Catheter and electrodes implantation

Surgical procedures were performed as described previously.^14,15^ Briefly, animals were anesthetized initially in 5% isoflurane (Piramal Healthcare, Digwal, Telangana, India) in air, and anesthesia was maintained with an intramuscular injection of medetomidine (Dormitor, 0.105 mg/kg body weight, Provet AG), midazolam (Dormicum, 1.4 mg/kg body weight, Roche), and fentanyl (0.007 mg/kg body weight, Kantonsapotheke Zürich).

After exposing the bladder, a PE-50 catheter was inserted through the bladder dome and secured with a purse-string suture. Two electromyography (EMG) electrodes were placed parallel to the urethra near the EUS, while a third ground electrode was sutured to the abdominal muscle. The catheter and electrodes were tunneled subcutaneously to the back of the neck and attached to a modified infusion harness (QC Single; SAI Infusion Technologies, Lake Villa, IL). No signs of urinary infections—e.g. cloudy urine—were observed during the time-course of the experiment.

### SCI

Animals were anesthetized initially in 5% isoflurane in air and maintained with an intramuscular injection of medetomidine, midazolam, and fentanyl. A T8 laminectomy was performed, and the dura was carefully removed. Thereafter, the dorsal three quarters of the spinal cord were transected bilaterally, resulting in a severe but incomplete injury. Analgesics (Rimadyl, 2.5 mg/kg body weight, Pfizer) and antibiotics (Bactrim, 15 mg/kg body weight, Roche) were applied immediately after surgery and daily during the first 14 post-operative days. After SCI, the bladder of the animals was emptied manually twice per day until the end of the experiment.

### Urodynamic and EUS-EMG measurements

Procedures were performed as described previously.^14,15^ Briefly, after placing the awake animal in a restrainer, saline was instilled into the bladder (120 μL/min), and intravesical pressure, released urine (weight), and EMG voltage were recorded simultaneously for at least three voiding cycles. The following urodynamic parameters were assessed: threshold pressure, maximum detrusor pressure, maximum detrusor pressure during storage phase, maximum flow rate, voided volume, voiding time, compliance, post-void residual, voiding efficiency, non-voiding contractions, and EUS-EMG.

To serve as a measurement of the rigidity of the bladder wall, bladder compliance was calculated as the intravesical pressure increase (threshold detrusor pressure minus baseline detrusor pressure) divided by the voided volume. Non-voiding contractions were defined as any increase in intravesical pressure more than 5 cmH_2_O that were not followed by urine flow. Quantification of high-frequency EUS-EMG activity was achieved by summing every high-frequency (20–500 Hz) power value in the period of interest—i.e., before, during, and after micturition—and dividing it by the sum of every high-frequency power value in the whole period analyzed.

Further analyses of the EUS-EMG data were based on the bursting period (defined as the total time where bursting was observed), active period (defined as the time during bursting in which the EUS-EMG was active), and silent period (defined as the time during the bursting period in which the EUS-EMG was not active).

### Tibial nerve stimulation

**FIG. 1.**
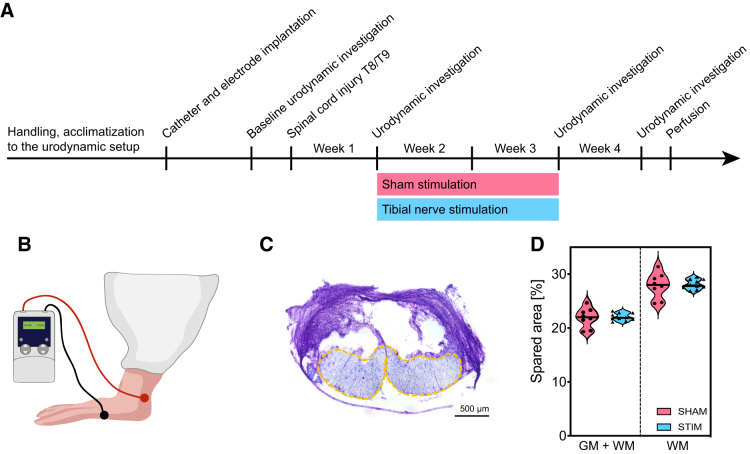
Investigation of transcutaneous tibial nerve stimulation effects on lower urinary tract dysfunction after an incomplete spinal cord injury. (**A**) Study timeline. (**B**) Location of the two stimulation electrodes on rat hindpaw. (**C**) Representative Nissl image of the lesion size, showing a spinal cord cross section at thoracic level 8/9. Yellow dashed-line shows the spared tissue. (**D**) Comparison of the spared area in relation to the whole spinal cord cross section (left) and to the white matter (right) for the two treatment groups. GM, gray matter; WM, white matter. Scale bar in (C) is 500 μm.

Starting at day 7 after SCI, all animals received either sham or verum stimulation for 15 consecutive days. Two modified self-adhering platinum electrodes (ø = 3 mm; Parsenn-Produkte AG, Switzerland) were placed slightly proximal and posterior to the medial malleolus and in the middle of the arch of the foot, respectively (Fig. 1B). Once the motor threshold was determined, the stimulation amplitude was reduced by 0.5 mA to reach a submotor level in the stimulation group, while the stimulator was completely turned off in the sham group.

All daily sessions were performed with a current frequency of 20 Hz, a pulse width of 200 μs, and a duration of 30 min. Stimulation parameters were chosen based on the frequency used for inhibition in the majority of the studies using tibial nerve stimulation,^13^ as well as the parallel clinical trial.^16^

### Tissue preparation

At the end of the study, rats were euthanized with an intraperitoneal overdose of pentobarbital (300 mg/mL, Streuli Pharma, Switzerland) and transcardially perfused with Ringer solution followed by 4% buffered paraformaldehyde (Sigma-Aldrich, Switzerland). Bladders were dissected, weighed, and stored at -80°C. Spinal cords were first post-fixed for 24 h at 4°C in the same fixative and then immersed in 30% sucrose in phosphate buffer.

Afterward, either 30- or 40-μm–thick cross sections of the lower thoracic (T7–T10) or lumbosacral (L5–S2) spinal cord, respectively, were cut on a cryostat. The bladder tissue samples were divided into two pieces. One half was snap frozen for protein analysis, while the other half was embedded in O.C.T. (optimal cutting temperature, Tissue-Tek) compound and cut in 8 μm-thick sections on a cryostat.

### Assessment of lesion completeness

Slides with 30 μm coronal sections of the lower thoracic (T7–T10) spinal cord were placed in increasing concentrations of ethanol (70%, 80%, 90%, 80%, 70%) for 30 sec each before being rinsed with water and incubated in cresyl violet (Sigma-Aldrich, Switzerland). After 5 min, sections were placed in increasing concentrations of ethanol (70%, 80%, 90%, 95%, 100%, 100%) for 30 sec each, followed by a final wash in xylene (90 sec), before being coverslipped with a mounting medium (Eukitt).

Cresyl violet images were used to reconstruct manually the maximal lesion extent in a spinal cord template based on a spinal cord atlas (*The Spinal Cord – A Christopher and Dana Reeve Foundation Test and Atlas, 2009*) using Adobe Illustrator CC 2020. The spared area compared with either the total cross section of the spinal cord or the total white matter was estimated using Fiji.^17^

### Bladder histology

The 8 μm-thick transverse sections were used to determine the amount of collagen and muscle present in the bladder after Masson trichrome staining (Sigma-Aldrich, Switzerland). The muscle to collagen ratio in the bladder was quantified by imaging five randomly chosen areas per animal with a bright-field microscope (10x; Zeiss, Axio Scan.Z1). Images were imported in Fiji, the region covered by each channel was thresholded, and the area calculated in %.

### Automated Western blot (WES)

Bladder tissues were pulverized in liquid nitrogen with a mortar/pestle and suspended in lysis buffer supplemented with a protease inhibitor cocktail (Sigma-Aldrich). Afterward, the samples were centrifuged for 20 min at 13,000 rpm, and the supernatant was collected for protein determination. Total protein was measured with the BCA Protein Assay Kit (Thermo Scientific, Lausanne, Switzerland).

For the WES sample preparation using 12–230 KDa cartridge kit, 1.2 mg/mL of protein was used, and the proteins were separated in WES with a capillary cartridge according to the manufacturer's protocol (Automated capillary western blotting, Protein Simple WES, Germany). The primary antibodies used for detection were mouse primary antibodies against alpha smooth muscle actin (αSMA) (Novus Biologicals, Centennial, CO, NBP2-33006, 1:100), calponin (Sigma-Aldrich, C2687, 1:400), and myosin heavy chain 11 (MYH11) (Santa Cruz, sc-6956, 1:10). Mouse anti-glyceraldehyde-3-phosphate dehydrogenase antibody (1:100, Novus Biologicals Europe) served as internal control.

### Immunofluorescence

Free-floating sections of the lumbosacral (L6–S1) spinal cord were blocked and permeabilized in TNB blocking solution containing 0.3% Triton-X and 5% normal goat serum for 60 min at room temperature, before being incubated with the primary antibody (rabbit-anti-calcitonin gene-related peptide [CGRP], 1:750, Millipore) diluted in TNB containing 0.05% Triton-X overnight at 4°C. The sections were then washed three time in 0.1 M phosphate-buffered saline (PBS) for 10 min each, incubated with secondary antibody (goat-anti-rabbit conjugated to Alexa Fluor 647; 1:500, Jackson ImmunoResearch Laboratories) for 2 h at room temperature, counterstained with 4',6-diamidino-2-phenylindole, and ultimately washed three times in 0.1 M PBS before being mounted on slides.

### Quantification of afferent CGRP-positive C-fibers

Three cross sections of the spinal levels L6 and S1 were picked randomly and imaged with a fluorescent microscope (20x; Zeiss, Axio Scan.Z1). Exposure time was optimized during the first imaging and kept constant across all sections. Mean gray values for the region of the dorsal horn were measured bilaterally for the three sections and averaged. Spatial densitometry analyses were performed by placing five rectangular shapes of 50 × 300 μm at the edge of the dorsal horn pointing toward the center and plotting their gray value profile. The values of the 10 single profiles were averaged to obtain a unique mean gray value profile per section.

### Statistical analysis

Animals were number-coded randomly, and investigators were blinded until the end of the analysis. Data in Table 1 are reported as means ± standard error of the mean (SEM), while graphs show the median and the interquartile range (plus min/max). For the comparison of the effect of tibial nerve stimulation on urodynamic parameters and CGRP-positive fiber profiles, a mixed-effect analysis followed by the Bonferroni *post hoc* correction was used to analyze the repeated measures. For comparison of bladder histology and composition, an unpaired, two-tailed *t* test was used. The value of significance was considered at *p* < 0.05. Statistical analysis and plotting of data were performed using Stata statistical software (Version 14, StataCorp) and GraphPad Prism 8 (GraphPad Software).

**Table 1.** Comparison of Urodynamic Parameters in Sham versus Stimulated Animals

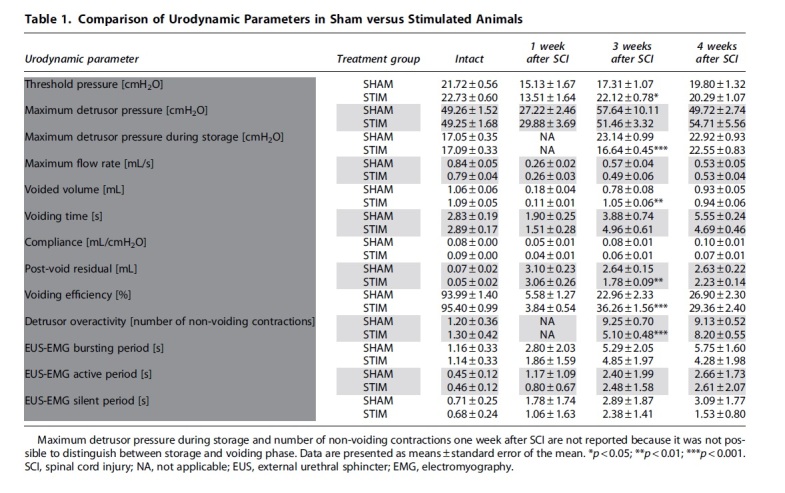

## Results

The transcutaneous tibial nerve stimulation protocol started one week after the spinal cord lesion with a daily 30 min session of continuous stimulation for a total of 15 days. Urodynamic investigations were performed before injury, at week 1 after injury before starting with the stimulation protocol, at week 3 after injury right after finishing with the stimulation, and finally at week 4—i.e., one week after the end of the tibial nerve stimulations (Fig. 1A,B). Immediately after injury, all animals showed a complete initial paralysis of the hindlimbs (data not shown). The thoracic microsurgical transection severed 77-81% of the total spinal cord cross section, interrupting 69-76% of the tracts running in the white matter (Fig. 1C–E). Lesion size was comparable between the sham and the stimulated groups.

Observations by eye of the animals during the experimental timeline did not indicate noticeable differences between the animals. At the end of the experiment, all animals showed some degree of hindlimb movement with occasional weight-supported steps, resembling a BBB score between 7 and 10. Two animals in the sham group were euthanized after the urodynamic investigation at one week after SCI because they did not recover from the injury, leaving eight animals in the sham group for weeks 3 and 4 after SCI.

**FIG. 2.**
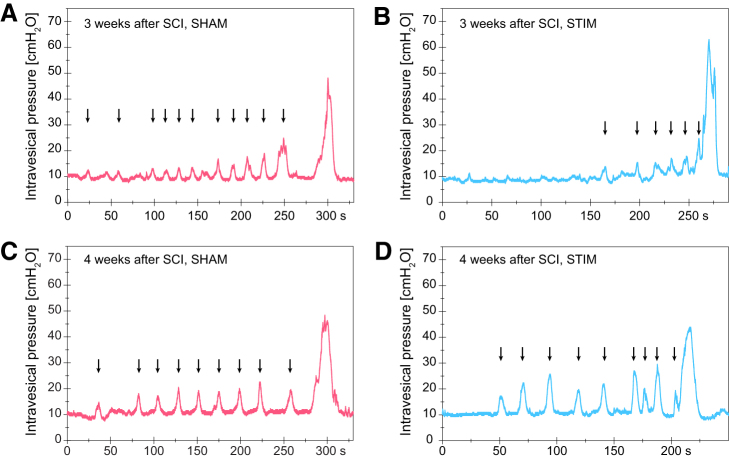
Urodynamic tracings following spinal cord injury (SCI). (**A,B**) Intravesical pressure during one representative micturition cycle at three weeks after injury—i.e., after the conclusion of the 15-day stimulation period in (A) sham and (B) stimulated animals. (**C,D**) Intravesical pressure during one representative micturition cycle four weeks after SCI—i.e., one week after the end of the stimulation period in (C) sham and (D) stimulated animals. Arrows indicate non-voiding contractions.

**FIG. 3.**
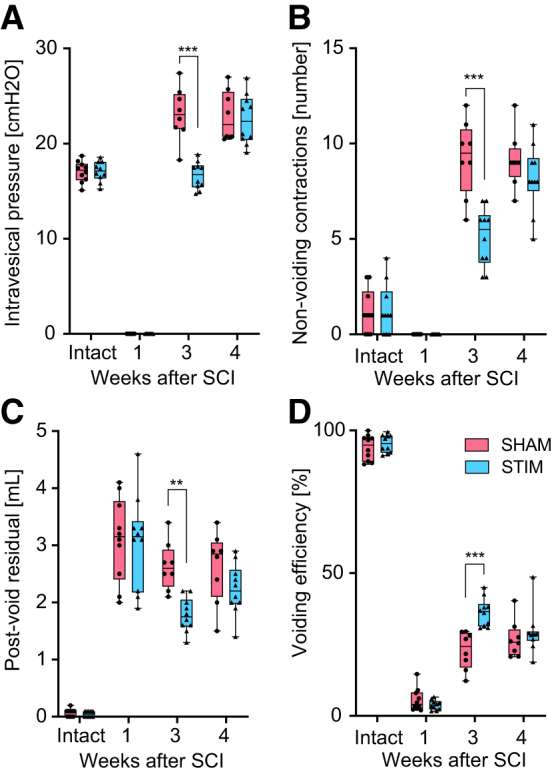
Intergroup differences in the urodynamic parameters over time in intact animals before spinal cord injury (SCI), one week after SCI before stimulation, three weeks after SCI, and after 15 days of stimulation, and four weeks after SCI and on week after stimulation. (**A**) Maximum intravesical pressure during urine storage. (**B**) Number of non-voiding contractions in a single micturition cycle. (**C**) Post-void residual measured at the end of the urodynamic investigation. (**D**) Voiding efficiency calculated by dividing the voided volume by the total bladder capacity. **p < 0.01; ***p < 0.00.

One week after SCI, all animals had urinary retention. Urodynamic investigations confirmed that the animals had an acontractile detrusor and displayed overflow incontinence during the examinations. Right after the end of the stimulation period (week 3), animals that received sham stimulation showed detrusor overactivity, as demonstrated by the increased number of non-voiding contractions and large rises in intravesical pressures during the storage phase (Fig. 2A, Fig. 3A, Table 1). In contrast, animals undergoing daily transcutaneous tibial nerve stimulation had fewer episodes of non-voiding contractions and a significantly lower maximum intravesical pressure during the storage phase (Fig. 2B, Fig. 3B, Table 1).

**FIG. 4.**
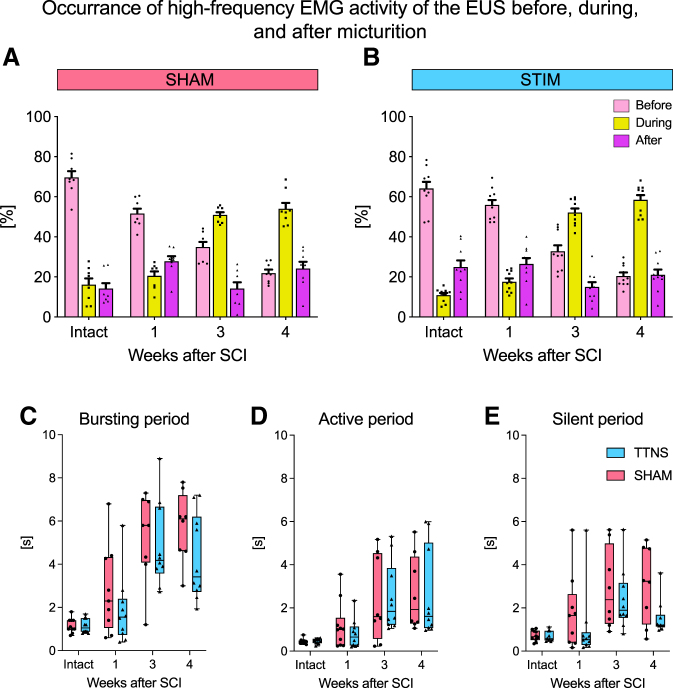
External urethral sphincter-electromyography (EUS-EMG) activity before and one, three, and four weeks after an incomplete spinal cord injury (SCI). (**A**) After a large but incomplete SCI, the high-frequency power activity (21–500 Hz) of the EUS in sham-stimulated animals shifts to the micturition phase starting three weeks after SCI, a condition typical for detrusor-sphincter-dyssynergia. (**B**) Stimulated animals showed a similar shift toward an increased EUS activity during micturition starting three weeks after SCI. The bursting activity of the EUS-EMG was further analyzed by assessing the (**C**) total bursting period, (**D**) active period, and (**E**) silent period. TTNS = transcutaneous tibial nerve stimulation.

In addition, stimulated animals had a significantly higher voided volume and a lower post-void residual, resulting in a higher voiding efficiency (Fig. 3C,D, Table 1). No differences were observed in maximum detrusor pressure and urinary flow rate. At three weeks after injury, symptoms of DSD developed in all animals as shown by an abnormal, high proportion of high frequency EMG during micturition, independent of the treatment group (Fig. 4A,B). No differences in the bursting activity of the EUS-EMG during voiding were observed between the two groups in any of the time points investigated (Fig. 4C–E). During the assessments conducted one week after the end of the stimulation period (week 4), we did not detect any differences in the lower urinary tract function between the two treatment groups anymore (Fig. 2C,D, Fig. 3A–D, Table 1).

**FIG. 5.**
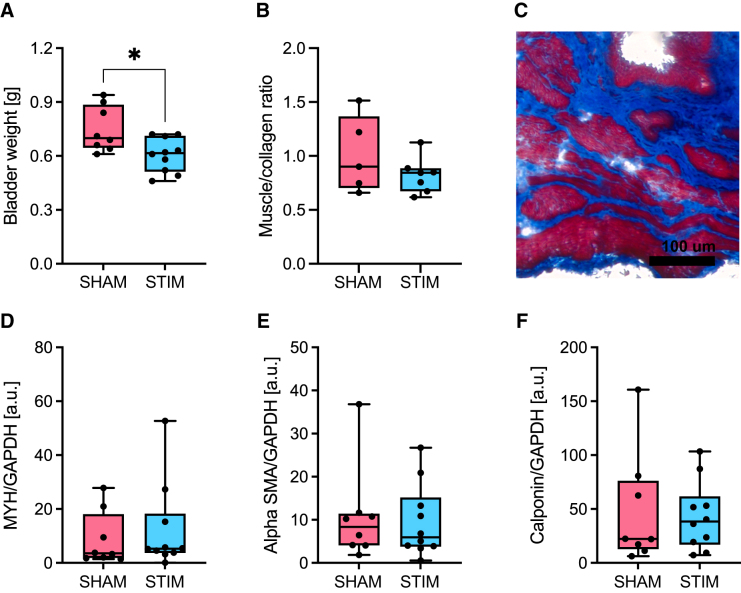
Assessment of bladder hypertrophy four weeks after spinal cord injury. (**A**) Bladder weight. (**B**) Quantification of the muscle to collagen ratio obtained by Masson Trichrome staining. (**C**) Representative Masson Trichrome staining of bladder tissue. Muscle is stained red, collagen blue. (**D–F**) Quantification of three muscle proteins by Western blot (WES): myosin heavy chain 11 (MYH11; 182 kDa), alpha-smooth muscle actin (SMA; 48 kDa), and calponin (45 kDa) in sham and stimulated groups. GAPDH, glyceraldehyde 3-phosphate dehydrogenase. *p < 0.05.

At the time of euthanasia, bladders were dissected and weighed. We observed that the unstimulated sham animals had a bigger and heavier—i.e. likely hypertrophic—bladder compared with animals that underwent tibial nerve stimulation (Fig. 5A). Histological analysis of bladder tissue revealed that, even though not statistically significant, sham animals had a tendency for a higher muscle to collagen ratio (Fig. 5B,C). Further analysis at the protein level by immunoblotting did not highlight any differences in the expression of smooth muscle contractile proteins αSMA, calponin, or MYH11 between the two groups (Fig. 5D–F).

**FIG. 6.**
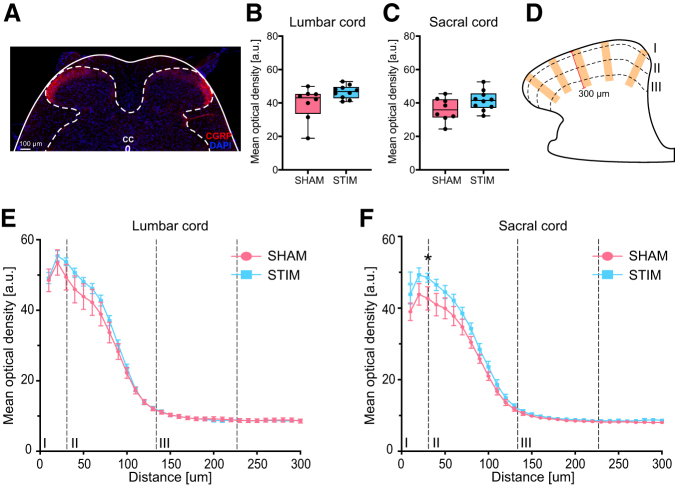
Assessment of C-fiber afferents in the lumbosacral dorsal horn. (**A**) Representative calcitonin gene-related peptide (CGRP) staining of the lumbosacral spinal cord (solid line). Dashed-line delimitates the gray matter. (**B,C**) Mean optical density quantification of CGRP in layers I–III in the (B) L6 and (C) S1 dorsal horn. (**D**) Schematic representation of the methods used to plot the CGRP profile over distance. Immunostaining intensity was determined in five rectangular shapes of 50 × 300 μm placed over laminae I-III in each dorsal horn. (**E,F**) Mean optical density quantification of CGRP immunofluorescence over distance from the most superficial portion of lamina I to the lower border of lamina III in (E) L6 and (F) S1 dorsal horns. Profiles are plotted in 10 μm intervals. Scale bar in (A) is 100 μm. *p < 0.05.

We further investigated the possible effects of tibial nerve stimulation on the density and spinal layer termination specificity of afferent C-fibers in the lumbosacral spinal cord. Using CGRP to label afferent fibers of type C (Fig. 6A), we observed a slightly higher density of CGRP-positive structures in layer I and II of the dorsal horn of L6 and S1 in the stimulated group (Fig. 6B,C,E,F). The layer specificity of the CGRP-positive fibers was very similar between the two experimental groups (Fig. 6E,F).

## Discussion

The loss of supraspinal inputs to the lumbosacral spinal cord after a large thoracic or cervical SCI leads to involuntary, reflexive detrusor contractions and, when the bladder filling is high, to simultaneous contraction of the detrusor and the external urethral sphincter (DSD). The DSD can cause persistent, elevated intravesical pressures that can induce vesicoureteral reflux, ultimately leading to kidney damage and failure. All these effects, well known in patients, were reproduced here in rats with SCI.

Transcutaneous tibial nerve stimulation daily over two weeks was feasible and well tolerated by the animals. Early application of stimulation one week after injury decreased the number of non-voiding bladder contractions, as well as the maximum intravesical pressure during storage. Stimulated animals were able to void more efficiently compared with the non-stimulated rats. The observed beneficial effects, however, were not long-lasting and vanished at one week after discontinuation of the neurostimulation. Importantly, tibial nerve stimulation did not hinder the development of DSD with the current stimulation protocol.

Recently, the focus of research in neuro-urology has shifted toward the direction of prevention of lower urinary tract dysfunction. Sievert and associates^18^ demonstrated that early application of sacral neuromodulation by implanted electrodes in patients with SCI was able to prevent the emergence of detrusor overactivity and related urinary incontinence. Recent studies on the much less invasive transcutaneous tibial nerve stimulation showed promising results in patients with neurogenic lower urinary tract dysfunction resulting from several neurological conditions.^19,20^

The effects of early application of tibial nerve stimulation have been investigated in a pilot study in patients with SCI. Interestingly, already 10 daily sessions of 30 min of submotor-threshold tibial nerve stimulation were effective in decreasing the development of the typical lower urinary tract dysfunction.^21^ This human finding is well in line with our observations in the present animal study at week 3 after severe thoracic SCI. Unfortunately, the human pilot study did not investigate whether the effects of the stimulation were maintained after cessation of the stimulations. With just 15 days of stimulation applied in the present rat study, the beneficial effects were only transitory.

Different and longer stimulation protocols—e.g., longer stimulation sessions, repeated over the day, longer total duration of the stimulation therapy—will have to be examined in future studies, as well as the value of periodically repeated stimulation sessions to maintain the treatment level. In fact, reinitiating tibial nerve stimulation after a period of time or starting it in patients with chronic SCI is certainly a very interesting aspect that was investigated recently by Kamboonlert and colleagues.^22^ They found that stimulation of patients with chronic SCI resulted in beneficial, acute effects on lower urinary tract dysfunction, opening new possibilities for this therapy.

Even though these clinical studies found improvements in DSD, our study showed an inefficacy of tibial nerve stimulation on preventing the development of DSD with this stimulation paradigm. Although this could result from a slightly different stimulation protocol, the methods to quantify DSD can highly affect the outcome as well. In fact, there is no standard procedure for EUS-EMG quantification, and most clinics define DSD either as present or absent and not depending on the power of the EUS-EMG. In our study, we used a semi-quantitative approach to quantify the EUS-EMG signal as well as parameters involved with the bursting activity of the EUS strictly associated with rodents. Unfortunately, none of these parameters showed a difference between the stimulated and sham groups.

It is important to notice that our study used a transection model to induce SCI, and this might not be the most translational model, because the majority of patients with SCI have a contusion injury.^23^ Nonetheless, we showed that the transection model produces reliable and very similar dysfunction as observed in patients with SCI, and the previous studies investigating the effects of different lesion types on lower urinary tract function did not highlight major differences,^24,25^ thereby making transection injury suitable for translational projects.

Bladder wall muscle hypertrophy is a common consequence of spinal cord lesion and the detrusor overactivity it induces.^3^ Although we did not detect differences in any of the urodynamic parameters investigated at the end of the experiment, the animals that received tibial nerve stimulation appeared to have a smaller and lighter bladder compared with sham animals. Bladder histological analysis showed a similar effect that, even though not statistically significant, points toward a positive effect of peripheral nerve stimulation on bladder structure.

Protein expression levels of αSMA, calponin, or MYH11 as detected by Western blotting were not different between the groups, however. Because the bladders were collected only one week after conclusion of tibial nerve stimulation, it is improbable that major anatomical differences could be observed between the end of the stimulation period and the collection of the bladders. The variability of these results was very large and may also be explained in part by disturbances in the detrusor caused by the implantation of the catheter.^15,26^

The bladder wall is densely innervated by sensory, pain receptive, CGRP-positive C-fibers. We therefore analyzed the density and potential remodeling of C-fiber afferents in the lumbosacral dorsal horn. These fibers are known to be involved in remodeling of the spinal micturition reflex pathway after injury.^27^ Contrary to intact conditions,^28^ in cats with chronic spinal cord injury, the micturition reflex could be initiated by activating C-fiber bladder afferents.^29^ Their involvement appeared to be crucial in eliciting detrusor overactivity, because administration of capsaicin, a C-fiber neurotoxin,^30^ reduced symptoms of detrusor overactivity.^31^

In the present rat study, we observed a slightly higher density of C-fibers in lamina I and II of the L6 or S1 dorsal horns of the stimulated animals. This tissue was collected at the end of the experiment, however, where differences in the urodynamic parameters between the groups were not detectable anymore; thus, the significance of these results remains unclear at present. Although previous literature hypothesized that tibial nerve stimulation effects might be from modulation of afferent pathways or neurotransmitter release at the level of the spinal cord,^29,32,33^ the data obtained from our animal study do not allow us to draw any conclusions. It would be ideal for future studies to investigate the activity induced by bladder afferent fibers at the level of the spinal cord using, for example, markers for immediate-early genes.

It is important to notice that the current animal study was initiated in combination with a clinical trial (ClinicalTrials.gov Identifier: NCT03965299) and tried to maintain the experimental design as close as possible to this clinical study. Yet, different stimulation parameters and protocols will have to be investigated in future trials,^34^ because they might lead to different outcomes. For example, Stampas and coworkers^21,35^ used a stimulation frequency of 10 Hz instead of 20 Hz used in our study, and this resulted in an improvement in DSD. This possibility is well in line with the current literature in animal research, which shows different outcomes based on a variety of stimulation parameters, including frequency.^36^

Two randomized clinical trials investigating the effects of tibial nerve stimulation in individuals with spinal cord injury are currently ongoing (ClinicalTrials.gov Identifier: NCT04350359 and NCT03965299) and will soon shed light on potential short- and long-lasting effects of this promising therapy^16^ applied in the first weeks after SCI. If they were to be successful, they could revolutionize the management of lower urinary tract dysfunction in patients with spinal cord lesions. In fact, this non-invasive therapy could be applied without interfering with other treatments in the rehabilitation centers as well as comfortably at the patient's home.

## Conclusions

Application of transcutaneous tibial nerve stimulation in rats with severe SCI early after lesion had a beneficial influence on the development of lower urinary tract dysfunction that typically arises after an incomplete SCI. Disappointingly, these beneficial effects disappeared one week after discontinuation of the stimulation. Improved stimulation protocols comprising longer or more intense stimulations, as well as maintenance treatments, will have to be investigated.

## Abbreviations Used

αSMA: alpha smooth muscle actin
CGRP: calcitonin gene-related peptide
DSD: detrusor-sphincter-dyssynergia
EMG: electromyography
EUS: external urethral sphincter
GAPDH: glyceraldehyde 3-phosphate dehydrogenase
MYH11: myosin heavy chain 11
PBS: phosphate buffered saline
SCI: spinal cord injury
TTNS: transcutaneous tibial nerve stimulation
WES: automated Western blot
